# Frequentist versus Bayesian approaches to multiple testing

**DOI:** 10.1007/s10654-019-00517-2

**Published:** 2019-05-13

**Authors:** Arvid Sjölander, Stijn Vansteelandt

**Affiliations:** 1 Karolinska Institutet, Nobels väg 12 A, 171 77 Stockholm, Sweden; 2 Ghent University, Krijgslaan 281, S9, 9000 Ghent, Belgium

**Keywords:** Bonferroni correction, Data fishing, Multiple comparisons, Multiple tests, Posterior distribution, *p*-value

## Abstract

Multiple tests arise frequently in epidemiologic research. However, the issue of multiplicity adjustment is surrounded by confusion and controversy, and there is no uniform agreement on whether or when adjustment is warranted. In this paper we compare frequentist and Bayesian frameworks for multiple testing. We argue that the frequentist framework leads to logical difficulties, and is unable to distinguish between relevant and irrelevant multiplicity adjustments. We further argue that these logical difficulties resolve within the Bayesian framework, and that the Bayesian framework makes a clear and coherent distinction between relevant and irrelevant adjustments. We use Directed Acyclic Graphs to illustrate the differences between the two frameworks, and to motivate our arguments.

## Introduction

Multiple tests (or comparisons) arise frequently in epidemiologic research. One common example is when a data set is stratified, and the exposure-outcome association is assessed separately within each stratum. Another example is when the exposure-outcome association is assessed for a range of different exposure variables. This happens, for instance, in genome-wide association scans (GWAS), in which there are often hundreds of exposure variables (e.g. SNPs). A third example is when a large set of competing models are fitted to the same data set, in search for the best model fit.

A well-known feature of multiple testing is that, as the number of tests increases, the number of ‘false findings’ tends to increase as well. For instance, if we stratify a data set finely, then the risk is large that we will obtain a significant *p* value in at least one stratum, even if the exposure and the outcome are truly independent in all strata.

Even though this feature is widely recognized, there is no uniform agreement on whether it is a problem that must be ‘handled’, or whether it is an innocent feature that may be ignored altogether when making inference. Kenneth Rothman, the founding editor of *Epidemiology* and one of the most influential and well-cited epidemiologists, took an extreme position when declaring that ‘No adjustments are needed for multiple comparisons’ [1]. A recent review article in the high impact journal *Nature Biotechnology* took the opposite extreme, by declaring that ‘in *any* experimental setting in which multiple tests are performed, *p* values must be adjusted appropriately [emphasis added]’ [2]. In our experience most researchers take a position somewhere in between these extremes, and the general notion seems to be that multiplicity adjustments are sometimes, if perhaps not always, warranted.

Nevertheless, multiplicity adjustments have not yet become part of epidemiological practice (except in GWAS studies, where they are commonplace). In an essay on multiple comparisons, Poole [3] recalled a situation where a group of (presumably experienced) statisticians was asked to suggest ways to contend with the problem of multiple testing. Poole writes ‘We responded by fidgeting slightly in our seats. When our disappointed leader asked if any of us had made any multiple-comparisons adjustments in the past five years or so, not a single hand went up. Someone finally broke the tension by joking about the distinction between theory and practice’. In our experience, this passage very accurately describes the current state of epidemiology; most epidemiological researchers approve of multiplicity adjustments in theory, but few use them in practice.

The reason why epidemiologists so rarely adjust for multiplicity cannot be that methods are lacking, since there is, in fact, an abundance of methods for multiplicity adjustments, of which many are implemented in standard software. Arguably, the reason cannot be perceived technical barriers either. Even though sophisticated and complex methods do exist, there are also very simple and non-technical methods (e.g. Bonferroni correction).

In our view, the reason is most likely that people find it logically difficult to distinguish between relevant and irrelevant multiplicity adjustments, and thus to determine a relevant collection of tests to adjust for. For instance, should a particular test be adjusted for all other tests in the same table, or for all tests in the same paper, or perhaps even for all tests that were made in the scientific process that eventually led up to the published paper? The literature gives no firm guidance; among all papers on multiplicity adjustments, the overwhelming majority deals with the technicalities of how to do statistically efficient adjustments, assuming that we have already agreed upon a relevant collection of tests to adjust for.

Berry and Hochberg [4] proposed a Bayesian framework for multiple testing. They showed that the logical difficulties of multiple testing resolve within their Bayesian framework, by assuming a joint a priori distribution on the (parameters of the) hypotheses being tested. Gelman et al. [5] made similar developments and conclusions. Despite its elegance, this Bayesian framework does not appear to be widely recognized by epidemiologists. The paper by Berry and Hochberg [4] has, according to Google scholar, fewer than 100 citations, of which most are in methodological statistics journals and none is in any of the leading epidemiological journals, e.g. *European Journal of Epidemiology*, *Epidemiology*, *International Journal of Epidemiology* or *American Journal of Epidemiology*.

The aim of our paper is to bring attention to the Bayesian framework by Berry and Hochberg [4], and to explain how it fundamentally differs from the frequentist framework. We start by giving a motivating example from a recently published study by Bygren et al. [6], which in many aspects is ‘typical’ for epidemiologic research. We use this example to illustrate how the frequentist framework leads to logical difficulties, in the context of multiple testing. We then review the Bayesian framework by Berry and Hochberg [4]. For pedagogical purposes we first restrict attention to tests on independent data sets; the case of dependent data sets is discussed in a separate section. We next show how the Bayesian framework makes a clear and coherent distinction, in principle, between relevant and irrelevant multiplicity adjustments.

An important case of multiple testing occurs when the researcher tests a large number of hypotheses and only reports those for which data happen to look ‘favorable’; this is often referred to as ‘data fishing’. We discuss data fishing in a separate section, and we argue that data fishing, surprisingly, does not require any special multiplicity adjustment within the Bayesian framework.

The main purpose of our paper is to provide conceptual insight. However, our conclusions do have strong practical implications, which we discuss in a separate section of the paper. In this section we return to the motivating example by Bygren et al. [6], and discuss how the results from this study may be viewed in light of the Bayesian framework.

The problem of multiplicity is usually discussed within the hypothesis testing paradigm, and most solutions that have been proposed (e.g. Bonferroni correction) are designed for *p* values. In line with this tradition we frame our exposition within the hypothesis testing paradigm as well. However, we note that this paradigm is often rightly criticized for conflating the effect size with the precision of the effect estimate [7], and that the effect estimation paradigm may often be more relevant for substantive epidemiologic research questions. We discuss effect estimation in a separate section, and show how the Bayesian framework applies within that paradigm.

Throughout, we use Directed Acyclic Graphs (DAGs) to motivate our arguments, and we assume that the reader has some familiarity with these. We refer to Pearl [8] and Greenland et al [9] for gentle introductions to DAGs.

## The frequentist framework: a motivating example

Bygren et al. [6] aimed to study whether change in food supply during grandparents’ early life may influence cardiovascular mortality in their grandchildren; a so-called ‘epigenetic effect’. Their sample consisted of 277 unrelated subjects from north of Sweden, and their grandparents. In their main analysis, the authors stratified the sample into eight groups, defined by each possible combination of grandparent (paternal grandfather, paternal grandmother, maternal grandfather, or maternal grandmother) and sex of the grandchild (male or female). The association between grandparent’s change in food supply and grandchild’s cardiovascular mortality was analyzed separately in these eight strata, with Cox proportional hazards regression. The results were presented as estimated hazard ratios, comparing the rate of cardiovascular mortality in exposed (to a change in food supply in the particular type of grandparent) and unexposed grandchildren, together with 95% confidence intervals; we have reproduced the results in Table 1. The authors did not report *p* values; however, we calculated these ‘post-hoc’ from the estimated hazard ratios and confidence intervals (see “Appendix 1” for details) and added them to Table 1.

**Table 1:** Estimated hazard ratios with 95% confidence intervals and *p* values, from Bygren et al. [6]

	Male			Female		
	$\widehat{\text {HR}}$	95% CI	*p*	$\widehat{\text {HR}}$	95% CI	*p*
Paternal grandfather	0.87	0.46–1.64	0.67	0.91	0.43–1.96	0.81
Paternal grandmother	0.64	0.32–1.29	0.21	**2.69**	**1.05–6.92**	**0.04**
Maternal grandfather	1.26	0.68–2.34	0.46	1.32	0.58–3.04	0.51
Maternal grandmother	0.69	0.35–1.36	0.28	0.56	0.22–1.49	0.22

Among the eight strata, only one finding was statistically significant (at 5% significance level); the hazard ratio in the stratum of paternal grandmother and female grandchild (bold in Table 1). Bygren et al. [6] took this single significant finding at face value, without making any multiplicity adjustment, and declared that ‘Change in paternal grandmothers early food supply influenced cardiovascular mortality of the female grandchildren’ (the title of the paper).

The paper caused some debate in Sweden, and was in particular heavily criticized by a Swedish professor in mathematical statistics, Olle Häggström, who insisted that the authors should have adjusted for multiplicity, and that not doing so was ‘bad practice’ [10]. Häggström’s critique was based on the standard frequentist argument that, if you test many null hypotheses, then you are likely to obtain at least one statistically significant *p* value, even if all null hypotheses are truly correct. Thus, the argument implies, in order not to be mislead by false positives one should adjust for the total number of tests.

The problem with this frequentist argument is not mathematical, but logical; how should we define the ‘total number of tests’? That is, what collection of tests should we consider when doing the multiplicity adjustment? All tests in the table? All tests in the published paper? All tests that were made in the scientific process that eventually led up to the published paper, even those that were not eventually included in the publication? All test in the issue of the journal? All tests ever published by that journal? And so forth... Even though our ‘gut feeling’ may point towards either of these alternatives, there is no way that we can use the frequentist argument to prove, or even indicate, why one alternative would be more relevant than another, since the argument can be applied equally well to any collection of hypothesis tests.

To further emphasize the logical problem, we note that Bygren et al. [6] actually carried out 16 more tests. In a secondary analysis, they additionally stratified the data by type of food supply pattern (poor to good or good to poor). They only observed a statistically significant result in the stratum of paternal grandmother, female grandchild and good to poor food supply. This type of secondary analysis is common in epidemiologic research; when an association is observed in a group of subjects one often proceeds by stratifying into finer subgroups, to investigate if the association is driven by any particular feature or covariate pattern.

To illustrate how the conclusions by Bygren et al. [6] are presumably flawed, Häggström [10] carried out a ‘post-hoc’ Bonferroni correction of their results. In this correction, he considered a total number of $8+16=24$ tests, thus implying that the main analysis should be adjusted for the number of tests in the secondary analysis as well. However, following this strategy the main analysis becomes increasingly more penalized as the number of secondary tests increases. A consequence is that, regardless how significant a result appears in a main analysis, one can always turn this result non-significant by simply carrying out a sufficiently large number of secondary tests, potentially unrelated to the test in the main analysis. Obviously, this does not seem reasonable. On the other hand, the distinction between ‘main’ and ‘secondary’ analyses is often a ‘thought-construction’, made up by the intention of the researcher. Thus, if we make a clear-cut between ‘main’ and ‘secondary’ analyses, and insist that tests within the former category should not be adjusted for tests within the latter category, then we allow for the intention of the researcher to affect the interpretation of the results. A consequence is that two researchers could claim different evidence with the same data, which seems unreasonable as well.

The problem of multiplicity adjustments is logically difficult, and we don’t claim that it can be solved in a purely algorithmic fashion by mathematical arguments. Regardless of what statistical framework one adheres to there is likely to be a gray zone, in which decisions have to be guided by subject matter knowledge and well founded opinion. However, what is deeply disturbing about the frequentist framework is that it seems unable to even indicate the underlying principles for these decisions. From the frequentist perspective all possible collections of tests seem equally valid to adjust for, and thus, any choice between these seems to be completely arbitrary.

## The Bayesian framework for independent data

In this section we review the Bayesian framework by Berry and Hochberg [4]. We note that our formulation differs slightly from Berry and Hochberg [4], in that these authors focused on effect estimation whereas we are mainly concerned with hypothesis testing.

To fix ideas we return to Table 1 and, for pedagogical purposes, only consider the strata with paternal grandmothers, i.e. the second row in Table 1. The two strata on this row consist of independent females and males. Let $\theta _f$ and $\theta _m$ be parameters indicating whether epigenetic effects are present for the females and males, respectively; $\theta _j=1$ for ‘effect present’ and $\theta _j=0$ for ‘effect absent’ in stratum *j*, for $j=f,m$. Thus, $\theta _j$ indicates whether the null ($\theta _j=0$) or alternative ($\theta _j=1$) hypothesis holds within stratum *j*.

**Fig. 1:**
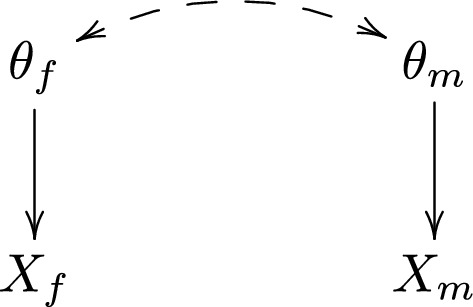
DAG for the Bygren et al. [6] study

In the Bayesian framework, $\theta _f$ and $\theta _m$ are considered random, and the relevant information about these is captured by their posterior distribution. To formalize, let $X_f$ and $X_m$ be the observed data for the females and males, respectively. These could be the raw data, but could also be functions of data, such as test statistics or *p* values. The DAG in Fig. 1 illustrates the relationship between $\theta _f$, $\theta _m$, $X_f$ and $X_m$. The arrow from $\theta _j$ to $X_j$ illustrates that the distribution of data in stratum *j* depends on whether the null or alternative hypothesis is true in stratum *j*, for $j=f,m$. The dashed double-headed arrow between $\theta _f$ and $\theta _m$ represents an a priori association between these parameter; we will see below that this association is a crucial component for multiplicity adjustment. The absence of a dashed double-headed arrow between $X_f$ and $X_m$ is motivated by the fact that the two strata consist of independent data.

A Bayesian analysis of the Bygren et al. [6] data would start by specifying an a priori distribution $p(\theta _f,\theta _m)$, and a probability model $p(X_f,X_m|\theta _f,\theta _m)$ for how data are distributed under the null and alternative hypotheses, respectively. The a priori distribution $p(\theta _f,\theta _m)$ may be specified explicitly, or may be derived from (the hyperparameters of) a hierarchical Bayesian model. Under the data generating mechanism in Fig. 1, $p(X_f,X_m|\theta _f,\theta _m)$ factorizes into $p(X_f|\theta _f)p(X_m|\theta _m)$. The a priori distribution $p(\theta _f,\theta _m)$ does not factorize, unless we assume that $\theta _f$ and $\theta _m$ are independent. Inference is based on the posterior distribution

1 $$\begin{aligned} p(\theta _f,\theta _m|X_f,X_m)= &  \frac{ p(X_f, X_m|\theta _f,\theta _m)p(\theta _f,\theta _m)}{\sum _{\theta _f,\theta _m=0,1}p(X_f, X_m|\theta _f,\theta _m)p(\theta _f,\theta _m)}\nonumber \\= &  \frac{p(X_f|\theta _f)p(X_m|\theta _m)p(\theta _f,\theta _m)}{\sum _{\theta _f,\theta _m=0,1}p(X_f|\theta _f)p(X_m|\theta _m)p(\theta _f, \theta _m)}. \end{aligned}$$

To carry out a Bayesian hypothesis test for the female stratum we marginalize over $\theta _m$ and compute the posterior probability of the alternative hypothesis:

2 $$\begin{aligned} p(\theta _f=1|X_f,X_m)=\frac{\sum _{\theta _m=0,1} p(X_f|\theta _f=1)p (X_m|\theta _m)p(\theta _f=1,\theta _m)}{\sum _{\theta _f,\theta _m=0,1}p(X_f| \theta _f)p(X_m|\theta _m)p(\theta _f,\theta _m)}. \end{aligned}$$

For instance, if the posterior probability is greater than 0.5, then the alternative hypothesis is more probable than the null hypothesis, so it would be rational to believe in the alternative, i.e. to estimate that $\theta _f$ is equal to 1. This procedure generalizes easily to scenarios with more than two tests.

Now, note from (2) that the posterior distribution for $\theta _f$ generally depends on the data observed in the male stratum, $X_m$. This dependency comes from the a priori association between $\theta _f$ and $\theta _m$; by observing the data $X_m$ we learn something about $\theta _m$, which in turn informs us about $\theta _f$. The a priori association between $\theta _f$ and $\theta _m$ represents a belief that, if epigenetic effects are present/absent for males, then they are likely to be present/absent for females as well, and vice versa. This belief could for instance be motivated by biological arguments; since males and females have very similar physiology, we may consider it unlikely that epigenetic effects are only present/absent for one of the sexes.

By specifying an a priori association between $\theta _f$ and $\theta _m$, the posterior estimates of these parameters thus ‘shrink’ towards a common value; 0 or 1. If the data in the male stratum, $X_m$, provide evidence for the alternative hypothesis in the male stratum ($\theta _m=1$), then it implicitly also provides evidence for the alternative hypothesis in the female stratum ($\theta _f=1$), through the posterior distribution for $\theta _f$. The strength of this ‘implicit’ evidence depends on the a priori association between $\theta _f$ and $\theta _m$; the stronger the association, the stronger the evidence. Thus, the Bayesian test for $\theta _f$ takes into account, not only the existence of second test (i.e. the test for $\theta _m$), but also the data available for that second test. When this has been taken into account, there is no rationale for making further multiplicity adjustment due to the existence of the second test, since all relevant information about $\theta _f$ is captured by its posterior distribution.

## The Bayesian framework for dependent data

When introducing the Bayesian framework in the previous section, we restricted attention to tests on independent data sets. In practice, data sets are often dependent. An extreme case occurs when the same group of subjects are analyzed multiple times, as in the study by Bygren et al. [6]. For each of the two columns in Table 1, the outcome (cardiovascular mortality) is analyzed in relation to four different ‘exposures’ (type of grandparent), but these four analyses are made on the same subjects (all males and all females in the sample, respectively).

**Fig. 2:**
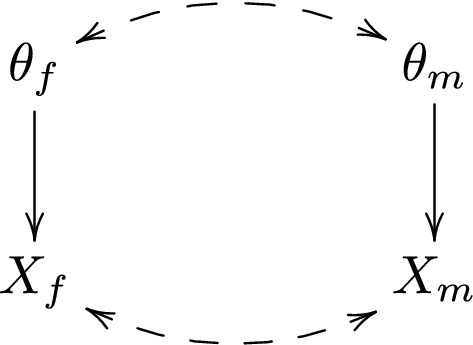
DAG for tests on dependent data sets

To see how the Bayesian framework applies to dependent data sets, consider the DAG in Fig. 2, in which the dashed double-headed arrow between $X_f$ and $X_m$ represents a ‘residual’ association between $X_f$ and $X_m$, which is not due to the a priori association between $\theta _f$ and $\theta _m$. In the presence of this arrow, $p(X_f,X_m|\theta _f,\theta _m)$ no longer factorizes into $p(X_f|\theta _f)p(X_m|\theta _m)$ as in (1). Thus, the posterior probability of $\theta _f=1$ may be written as

$$\begin{aligned} p(\theta _f=1|X_f,X_m)= &  \frac{\sum _{\theta _m=0,1} p(X_f,X_m| \theta _f=1,\theta _m)p(\theta _f=1,\theta _m)}{\sum _{\theta _f,\theta _m=0,1} p(X_f,X_m|\theta _f,\theta _m)p(\theta _f,\theta _m)}, \end{aligned}$$

but does not simplify further as in (2).

In principle, this modification does not pose difficulties for the Bayesian framework. However, it has important, and somewhat counter-intuitive, implications for multiple testing. Note that, under the DAG in Fig. 2, the conditional association between $\theta _f$ and $X_m$, given $X_f$, arises from two paths; $\theta _f\leftarrow \rightarrow \theta _m\rightarrow X_m$ and $\theta _f\rightarrow X_f\leftarrow X_m$. The path through $\theta _m$ is due to the a priori association between $\theta _f$ and $\theta _m$ discussed in the previous section. The path through $X_f$ is due to the ‘residual’ association between $X_f$ and $X_m$; this path becomes open by conditioning on the ‘collider’ $X_f$ [11]. If the $\theta _f$-to-$X_f$ association and the ‘residual’ $X_f$-to-$X_m$ association have the same direction (e.g. positive), then the open path through $X_f$ would generally tend to make $\theta _f$ and $X_m$ negatively associated. To see this, note that when both associations are positive, a large value of $X_f$ can be ‘explained’ by either a large value of $\theta _f$ or a large value of $X_m$, or both. If, in fact, we observe a small value of $\theta _f$, then this ‘explanation’ is ruled out, so that the other ‘explanation’—a large value of $X_m$-becomes more likely. That is, conditional on (a large value of) $X_f$, $\theta _f$ and $X_m$ are generally negatively associated. By symmetry we also have that, if the $\theta _f$-to-$X_f$ association and the ‘residual’ $X_f$-to-$X_m$ association have opposite directions, then the open path through $X_f$ would generally tend to make $\theta _f$ and $X_m$ positively associated.

To see that this behavior may have counter-intuitive implications, suppose that $X_f$ and $X_m$ are the *p* values in the female and male stratum, respectively, so that $\theta _f$ and $X_f$ are negatively associated (the alternative hypothesis $\theta _f=1$ is associated with a small *p* value $X_f$). Suppose further that $X_f$ and $X_m$ are positively associated, given ($\theta _f,\theta _m$). Suppose finally that $\theta _f$ and $\theta _m$ are (assumed to be) positively associated as well. A small *p* value $X_m$ indicates that $\theta _m=1$, which we would expect to indicate that $\theta _f=1$. However, according to the argument above, the path through $X_f$ would tend to make $\theta _f$ and $X_m$ positively associated. Thus, if the path through $X_f$ is ‘stronger’ than the path through $\theta _m$, then a small *p* value $X_m$ would instead indicate that $\theta _f=0$. In “Appendix 2”, we confirm this counter-intuitive behavior with a simulation.

In practice, the ‘net’ association between $\theta _f$ and $X_m$ depends on both the path through $\theta _m$ and the path through $X_f$, which may pull the association between $\theta _f$ and $X_m$ in different directions. It is difficult to give any general criteria for when either of these paths is likely to be stronger than the other.

## Relevant and irrelevant multiplicity adjustments

We have argued that people often find it logically difficult to distinguish between relevant and irrelevant multiplicity adjustments, and thus to determine a relevant collection of tests to adjust for. We have criticized the frequentist framework for not giving any guidance in this matter. What, then, does the Bayesian framework suggest?

Let’s first establish that certain adjustments are completely irrelevant in the Bayesian framework. To simplify language we say that two tests are independent if both the hypotheses and the data sets of the tests are independent, e.g. if both dashed double-headed arrows in Fig. 2 are absent. Conversely, we say that two tests are associated if either the hypotheses, or the data sets, of the tests are associated, e.g. if either dashed double-headed arrow in Fig. 2 is present. If the tests of $\theta _f$ and $\theta _m$ are independent, then $p(\theta _f,\theta _m)$ factorizes into $p(\theta _f)p(\theta _m)$, so that the posterior probability of $\theta _f=1$ in (2) simplifies further to

$$\begin{aligned} p(\theta _f=1|X_f,X_m)= &  \frac{\sum _{\theta _m=0,1} p(X_f|\theta _f=1)p(X_m|\theta _m)p(\theta _f=1)p(\theta _m)}{\sum _{\theta _f, \theta _m=0,1}p(X_f|\theta _f)p(X_m|\theta _m)p(\theta _f)p(\theta _m)}\\=\, &  \frac{ p(X_f|\theta _f=1)p(\theta _f=1)}{\sum _{\theta _f=0,1}p(X_f| \theta _f)p(\theta _f)}\\=\, &  p(\theta _f=1|X_f). \end{aligned}$$

This is the posterior probability of $\theta _f=1$ when the male stratum is completely ignored. That is, if the two tests are independent, then the male stratum is completely uninformative about the (hypotheses of the) female test. Thus, we arrive at the following important conclusion:

**A: It is irrelevant to adjust one test for another test, if the two tests are independent.**

This conclusion may be provocative, as it may seem to invite ‘data-fishing’, i.e. to test a large number of hypotheses and only report those for which data happen to look ‘favorable’. We discuss this issue in the next section.

What adjustments are then relevant? Within the Bayesian framework it is relevant to ‘adjust’ for all information that influence the posterior probability of the hypothesis under consideration. In the previous sections we have shown that tests carry mutual information if the hypotheses and/or the data sets are associated. Thus, we conclude:

**B: It is relevant to adjust one test for another test, if the two tests are dependent.**

Conclusion B has far-reaching implications. Consider the rhetorical question that we phrased in the Introduction: ‘should a considered test be adjusted for all other tests in the same table, or for all tests in the same paper, or perhaps even for all tests that were made in the scientific process that eventually led up to the published paper?’ Following conclusion B above, the answer is: ‘all of these adjustments are relevant! ... provided that all these tests are associated with the considered test.’ One may then play the devil’s advocate and ask ‘why draw the line at the published paper? Why not demand that adjustment is made for all tests in the world that are associated with the considered test?’ Indeed! From a Bayesian perspective all these adjustments are relevant, in the sense that all these tests are informative about (i.e. influence the posterior probability of the hypotheses of) the considered test.

We emphasize that this demand is, in principle, not at all unreasonable. It just means that any serious researcher should view his/her finding in light of others’ findings, as well as existing expert knowledge in the field. Of course, it is virtually impossible to adjust for ‘all associated tests in the world’ in a formal Bayesian analysis. We return to this issue in section ‘Practical implications’, where we propose a more informal approach.

## Data fishing and the intention of the researcher

Consider the following, somewhat provocative, example. Suppose that we invent 20 rather dubious, but unrelated, hypotheses, e.g. ‘milk causes cancer’, ‘red clothes cause cancer’, ‘being born under a certain sign of the zodiac causes cancer’ etc. Suppose that we compute one posterior probability for each hypothesis, on independent data sets. Suppose further that only the posterior for the ‘milk causes cancer’ hypothesis is large, e.g. $>0.5$. According to conclusion A in the previous section, it is then perfectly sound and rational to ignore the other 19 tests when interpreting this ‘finding’, and declare milk as a likely cause of cancer.

What may be disturbing with this example is that we didn’t specify in advance what hypothesis to report evidence for. In our particular realization of data, the posterior for ‘milk causes cancer’ turned out to be $>0.5$, so we chose to report that. However, if we were to repeat the 20 studies, then another (set of) posterior(s) may turn out to be $>0.5$, so that the ‘finding’ moves around over the 20 hypotheses. Thus, it may seem like our intention to selectively report evidence for the most favorable result invalidates the statistical inference, and people may accuse us for ‘data fishing’. A related example of how the intention of the researcher may play a role, is when the researcher collects data sequentially, with the intention to stop when data ‘look favorable’.

However, criticisms about data fishing have no bearing within the Bayesian framework. This is because the posterior probability of a particular hypothesis does not depend on whether we decided to report the evidence for this hypothesis in advance, or because the observed evidence happened to look favorable in retrospect. To better understand why this is case, consider again the example from Section ‘The Bayesian Framework for independent data’, where we tested two hypotheses indexed with parameters $\theta _f$ and $\theta _m$. Suppose that we have decided to pick cherries by only reporting the most ‘striking’ result, that is, we report the results for the female stratum if the posterior probability of $\theta _f=1$ is larger than the posterior probability of $\theta _m=1$, and vice versa. Note that these posterior probabilities are functions of data. In the spirit of *p* values (and with a slight abuse of notation), we may thus say that $X_f<X_m$ if $p(\theta _f=1|X_f,X_m)>p(\theta _m=1|X_f,X_m)$, and $X_f>X_m$ if $p(\theta _f=1|X_f,X_m)<p(\theta _m=1|X_f,X_m)$. We define a ‘selection variable’ *S*, which indicates what stratum we report the results for, as

$$\begin{aligned} S=\left\{ \begin{array}{lll}f & \text {if} & X_f<X_m\\ m & \text {if} & X_f>X_m\end{array}\right. \end{aligned}$$

**Fig. 3:**
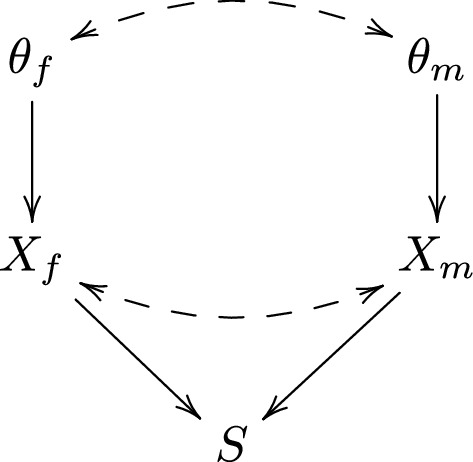
DAG for data fishing selection mechanism

The DAG in Fig. 3 illustrates the scenario. To be as general as possible, we have allowed for a ‘residual association’ between $X_f$ and $X_m$, as in Section ‘The Bayesian framework for dependent data’.

Suppose now that we observe that $X_f<X_m$, and accordingly choose to report the results for the female stratum. To acknowledge the selection process we could condition on $S=1$, thus reporting $p(\theta _f=1|X_f,X_m,S=f)$. However, in Fig. 3 we observe that $\theta _f$ is conditionally independent of *S*, given ($X_f,X_m$), so that $p(\theta _f=1|X_f,X_m,S=f)=p(\theta _f=1|X_f,X_m)$. Thus, the selection process is irrelevant within the Bayesian framework. The data $X_m$ are not irrelevant though when reporting the evidence for $\theta _f$, since $\theta _f$ and $X_m$ are associated through the paths $\theta _f\leftarrow \rightarrow \theta _m\rightarrow X_m$ and $\theta _f\rightarrow X_f\leftarrow X_m$. If these paths were absent (i.e. if the hypotheses were a priori independent and tested on independent data sets), then it would be perfectly sound to ignore $X_m$ as well, and report $p(\theta _f=1|X_f)$.

We thus conclude that, in the example above of 20 dubious but unrelated hypotheses above, it is perfectly sound to ignore the selection process when reporting the posterior for ‘milk causes cancer’. Furthermore, since we assumed that the 20 hypotheses were tested on independent data sets, it is also perfectly sound to ignore the data obtained for testing the other 19 hypotheses. Berger and Berry [12] made similar points, and argued more generally that, for Bayesian inference, the intention of the researcher is indeed irrelevant.

In contrast, the intention of the researcher does matter within the frequentist framework. To see this, let $X_f$ and $X_m$ be the *p* values for the female and male stratum, respectively. A pure frequentist would consider the distribution of the *p* value, and in particular require that the *p* value has a uniform distribution under the null hypothesis $\theta _f=0$. In Fig. 3 it is not true that $X_f$ is conditionally independent of *S*, given $\theta _f$. Thus, while $p(X_f|\theta _f=0)$ may be uniform, $p(X_f|\theta _f=0,S=f)$ is generally not.

We end this section by noting that, even if data fishing strategies do not require any multiplicity adjustments within the Bayesian framework, they are problematic for another reason. When researchers tend to under-report ‘non-findings’ the accumulated evidence in the field becomes skewed, which is often referred to as ‘publication bias’. We emphasize though, that this problem is unrelated to the issue of multiplicity, and that the publication bias due to those results that are not reported does not become less severe if we adjust the reported results for multiplicity.

## Practical implications

In previous sections we have compared frequentist and Bayesian frameworks for multiple testing. We have criticized the frequentist framework for leading to logical difficulties, and for being unable to distinguish between relevant and irrelevant multiplicity adjustments. We have argued that the Bayesian framework provides a clear and coherent distinction; within this framework it is only relevant to adjust for those tests that are associated with the test under consideration. Furthermore, the Bayesian framework provides a clear principle for *how* to adjust for multiplicity; by accounting for the multiple tests in the posterior probability. Finally we have argued that, in contrast to the frequentist framework, data fishing does not require any special adjustment within the Bayesian framework.

Despite these strong conceptual advantages, the Bayesian framework has important practical limitations. A formal implementation requires a quantitative specification of the a priori association between hypotheses. In practice it may be hard, even for subject matter experts, to put an exact number to this. Thus, a formal implementation of the Bayesian framework for multiple testing may, in our view, give a false sense of objectivity. We note though that this problem is not unique for multiple testing, but present in all types of Bayesian analyses.

A more severe problem is that the set of relevant adjustments may be extremely large. We concluded in Section ‘Relevant and irrelevant multiplicity adjustments’ that when considering a particular test it is, in principle, relevant to adjust for ‘all other tests in the world’ that are associated with the considered test. Clearly, it would be impossible to do this with a formal Bayesian analysis.

That said, we do feel that the Bayesian framework is appropriate for reasoning about multiplicity, as it ‘links together’ different tests in a natural way, without leading to any obvious logical difficulties. Thus, we propose a compromise, where the formal statistical analyses is done within the standard frequentist framework (e.g. by computing *p* values), and the adjustment for multiplicity is done informally, by reasoning qualitatively about the association of hypotheses.

To be concrete, let’s return to the study by Bygren et al. [6]. Table 1 presents one significant (< 0.05) and seven non-significant *p* values. When interpreting the single significant *p* value we should, according to the Bayesian framework, consider the ‘big picture’ and think about the a priori association between the hypotheses being tested in the eight strata of Table 1. Specifically, we should ask ourselves: ‘how likely is it that epigenetic effects are truly present in one stratum but absent in all other strata?’ The less likely we consider this scenario, the more ‘informal weight’ should be given to the seven non-significant *p* values when interpreting the one significant *p* value. We are not epigeneticists ourselves, and thus not in a position to speculate about a priori associations between hypotheses in this context. However, we note that the single significant *p* value was just below the traditional 5% significance level. A very modest association between hypotheses may suffice to throw this *p* value above the significance level, if properly corrected for the other *p* values through Bayes theorem. Thus, if we consider the 5% significance level as a relevant threshold for evidence (which can certainly be debated), then it seems unjustified to conclude that ‘change in paternal grandmothers’ early food supply influenced cardiovascular mortality of the female grandchildren’. However, if Bygren et al. [6] could argue convincingly that the hypotheses in Table 1 are truly independent, then their conclusion may indeed be reasonable, and not a result of ‘bad practice’, as claimed by Häggström [10].

In a similar fashion, a researcher may informally ‘adjust’ his/her results for evidence published in other papers (e.g. ‘all other tests in the world’). In practice, this means that the researcher may survey the field and identify those studies that have similar research questions and hypotheses as the researcher’s own study. If the results from these studies are coherent with the researcher’s own result, then the evidence is strengthened. If not, then the evidence is weakened. This informal procedure of accumulating evidence is already common practice, and often an important part of the ‘Discussion’/‘Conclusion’ section in applied epidemiological papers. Thus, the Bayesian framework provides a formal underpinning for common practice.

The Bayesian framework has important implications for how we should think about ‘data fishing’ and, more generally, about the intention of the researcher. In epidemiological studies, researchers typically focus on a limited number of hypotheses. Often, a distinction is made between ‘primary’ and ‘secondary’ hypotheses, where the emphasis if typically put on the former. If a large number of additional analyses have been done, then these are often described as more speculative (e.g. ‘exploratory’ or ‘hypothesis generating’). Arguably, a strong reason for this habit is that researchers want to avoid being accused for data fishing. In clinical trials, this is avoided by specifying the hypotheses and analysis plan in advance; notably, this has recently been advocated for observational studies as well [13, 14]. However, an important message of our paper is that, within the Bayesian framework, the intention of the researcher does not matter. Thus, if one adheres to the informal Bayesian adjustment for multiplicity outlined above, then accusations of data fishing have no bearing.

We note though that there could be other good reasons for limiting the number hypotheses within a given study, or distinguishing between ‘primary’ and ‘secondary’, such as improving transparency when presenting the results. We also note that the Bayesian framework does not give *carte blance* to test a large number of hypotheses, and selectively report the hypothesis with, say, the smallest *p* value, without showing the other *p* values as well. Showing all *p* values in Table 1 makes it possible for the reader to evaluate the one significant *p* value in light of the seven non-significant *p* values, by reasoning quantitatively about the association of hypotheses as outlined above. If only the one significant *p* value is presented, then relevant information is hidden from the reader. The important exception is when the tests are independent, as defined in Section ‘Relevant and irrelevant multiplicity adjustments’. In this case, no information is shared across tests, and it is fine to report the smallest *p* value alone. We note though that this would rarely be the case in real epidemiological studies, since the hypotheses being tested within a particular study are typically related.

We end this section by emphasizing that, in order for two tests to be associated, it is enough that the data sets are dependent. Thus, when data for (some of) the tests are dependent, as in Table 1, our proposal to reason qualitatively about the association of hypotheses only goes half-way. Furthermore, as we showed in Section ‘The Bayesian framework for dependent data’ it may not be obvious how evidence is propagated across tests. We acknowledge this as an important aspect of the Bayesian framework, and we hope that future research may give guidance on how to handle this problem in practice.

## Effect estimation

In line with most of the literature, we have framed our discussion on multiplicity within the hypothesis testing paradigm. However, the problem of multiplicity arises within the effect estimation paradigm as well. For instance, the probability of finding at least one estimate with a large magnitude increases with the number of estimated effects, even if all effects are truly small. The Bayesian framework can easily be used for effect estimation; indeed, the paper by Berry and Hochberg [4] focused on effect estimation rather than hypothesis testing.

As an illustration, consider again the example from Section ‘The Bayesian Framework for independent data’. To modify this example for effect estimation we would simply redefine the parameters $\theta _f$ and $\theta _m$ as the effects of interests, e.g. the hazard ratios in the female and male stratum, respectively. We would then, as before, specify an a priori distribution $p(\theta _f,\theta _m)$, and a probability model $p(X_f,X_m|\theta _f,\theta _m)=p(X_f|\theta _f)p(X_m|\theta _m)$ for how data are distributed under the true parameter values. In line with the analysis by Bygren et al. [6], we may use Cox proportional hazards models for $p(X_f|\theta _f)$ and $p(X_m|\theta _m)$, but other (semi)parametric models are possible as well. Inference is based on the posterior distribution in (1). For instance, to estimate the hazard ratio in the female stratum we would use the posterior distribution in (2); a natural estimate would be the mode or mean in this distribution. As for hypothesis testing, the posterior distribution for $\theta _f$ generally depends on the data observed in the male stratum, $X_m$, through the a priori association between $\theta _f$ and $\theta _m$. Thus, the posterior estimates of $\theta _f$ and $\theta _m$ are ‘shrunk’ towards each other. In this sense, the a priori distribution $p(\theta _f,\theta _m)$ accounts for multiplicity within the effect estimation paradigm, by giving appropriate weight to the data $X_m$ when estimating $\theta _f$, and vice versa.

## Discussion

The problem of multiple testing is prevailing in epidemiologic research. We foresee that this problem will become even more acute in the near future, with the increasing availability of rich and complex data sets. In this paper we have argued that the Bayesian framework resolves the logical difficulties of multiple testing, and that this framework provides a clear and coherent distinction between relevant and irrelevant multiplicity adjustments. We have further argued that, although the Bayesian framework provides strong conceptual clarity, a formal implementation may not be desirable. Thus, we have advocated a compromise, where the formal statistical analyses is done within the standard frequentist framework (e.g. by computing *p* values), and the adjustment for multiplicity is done informally, by reasoning qualitatively about the association of hypotheses. We note though that the Bayesian framework can be formally implemented with standard software. We provide an example with simulated data in “Appendix 3”.

## Figures and Tables

**Fig. 4:**
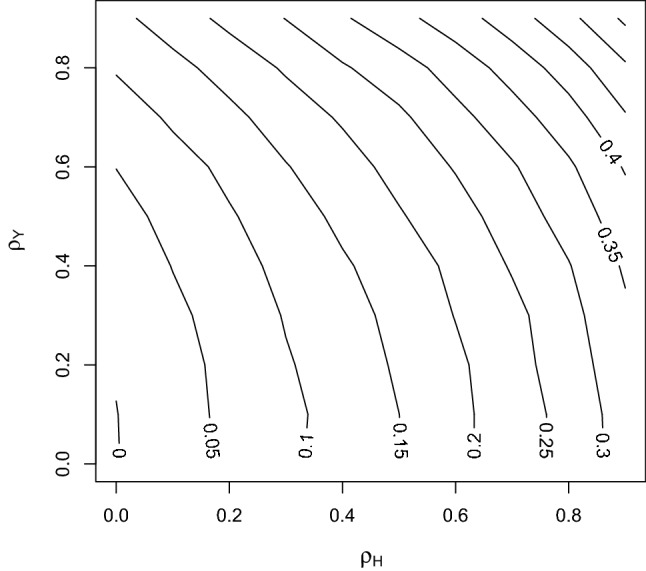
$cor(X_f,X_m)$ as a function of $(\rho _H,\rho _Y)$

**Fig. 5:**
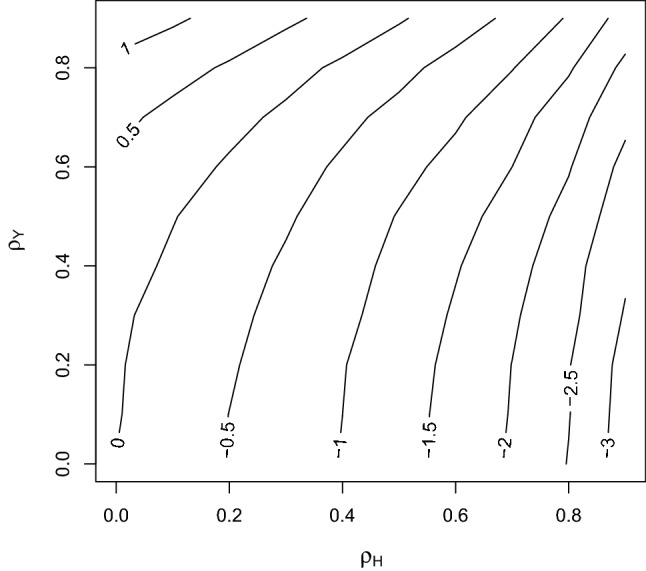
$\beta _m$ as a function of $(\rho _H,\rho _Y)$

**Fig. 6:**
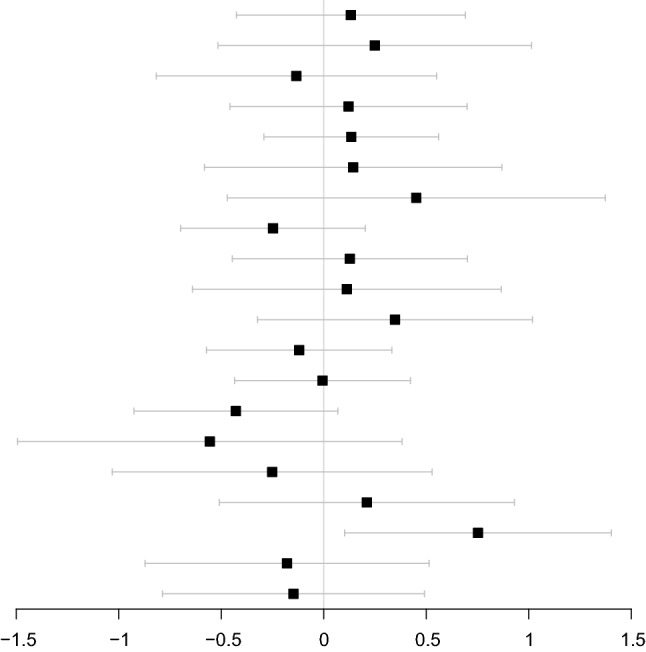
Frequentist estimates and 95% confidence intervals

**Fig. 7:**
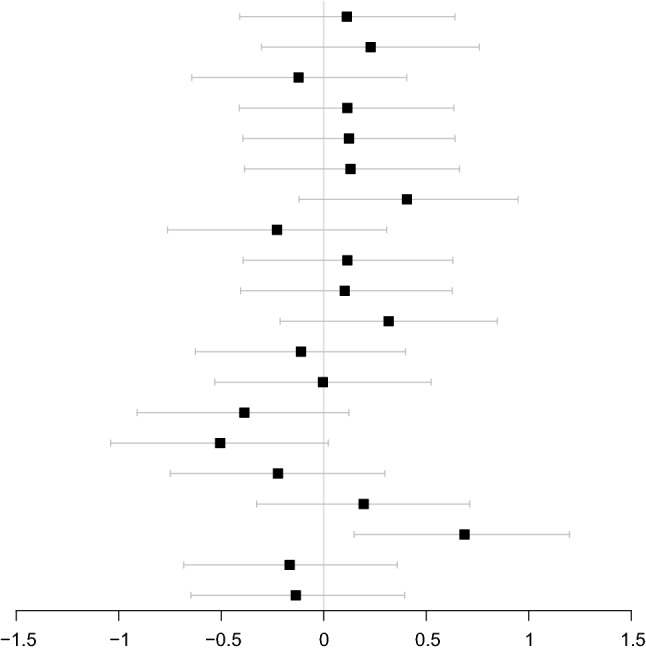
Bayesian estimates and 95% credible intervals, for independent elements of $\beta$

**Fig. 8:**
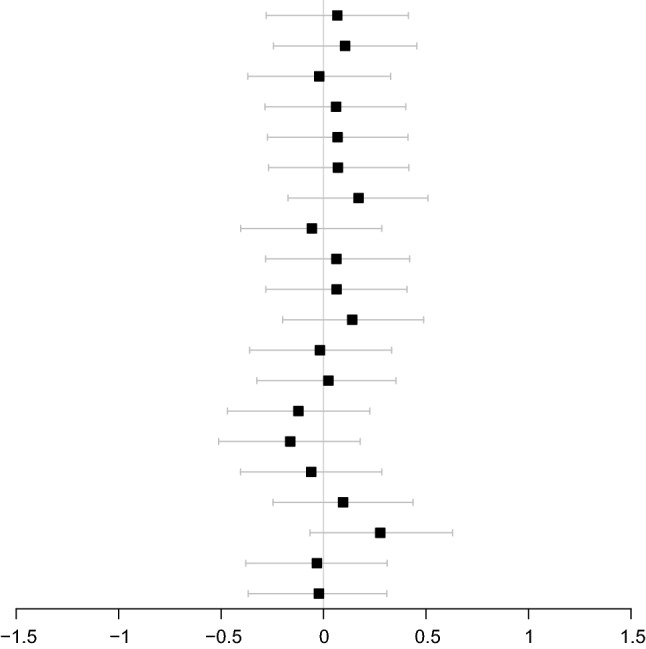
Bayesian estimates and 95% credible intervals, for strongly dependent elements of $\beta$, with $\text {cor}(\beta _j,\beta _k)=0.95$

**Fig. 9:**
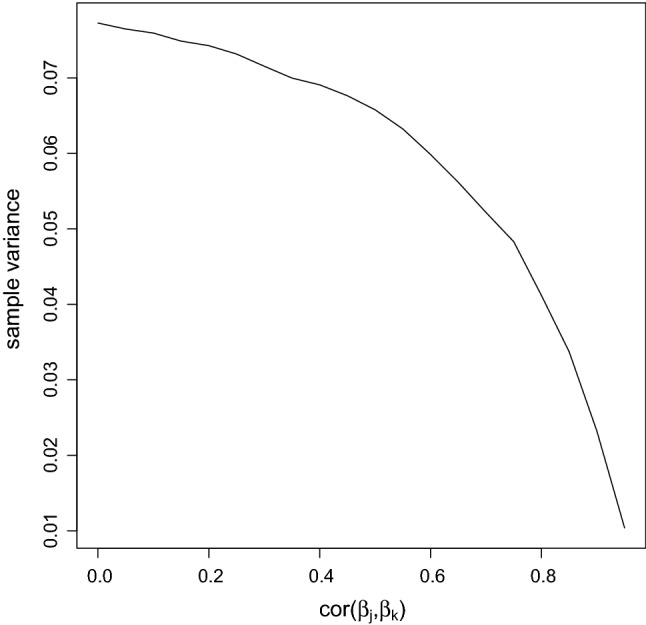
Sample variance of the estimates $(\hat{\beta }_1, \ldots , \hat{\beta }_J)$ as a function of $\text {cor}(\beta _j,\beta _k)$

## Appendix 1: Reconstruction of *p* values in Table 1

To reconstruct the *p* values in Table 1 we used the relation between a Wald confidence interval and a Wald test. The lower limit of a 95% Wald confidence interval for $\text {log}(\text {HR})$ is given by

$$\begin{aligned} \text {CI}_{\text {lower}}=\text {log}(\widehat{\text {HR}})-1.96\times s.e. \end{aligned}$$

from which we have that

$$\begin{aligned} s.e.=\frac{\text {log}(\widehat{\text {HR}})- \text {CI}_{\text {lower}}}{1.96}. \end{aligned}$$

The *p* value for a Wald test of the null hypothesis $\text {log}(\text {HR})=0$ is given by

3 $$\begin{aligned} p= &  2\left\{ 1-\Phi \left( \left|\frac{\text {log}(\widehat{\text {HR}})}{s.e.}\right|\right) \right\} \nonumber \\= \,&  2\left\{ 1-\Phi \left( \left|\frac{1.96\times \text {log}(\widehat{ \text {HR}})}{\text {log}(\widehat{\text {HR}})-\text {CI}_{ \text {lower}}}\right|\right) \right\} , \end{aligned}$$

where $\Phi (\cdot )$ is the standard normal distribution function. Plugging reported values of $\text {log}(\widehat{\text {HR}}$ and $\text {CI}_{\text {lower}}$ into (3) gave the *p* values in Table 1. Using the upper limit of the Wald confidence interval instead gave very similar *p* values.

## Appendix 2: Simulation with dependent data

To simulate hypotheses that are correlated across tests we generated two ‘latent’ parameters $\theta ^*_f$ and $\theta ^*_m$ from

$$\begin{aligned} \left( \begin{array}{c} \theta ^*_f\\ \theta ^*_m\end{array} \right)\sim &  N\left\{ \left( \begin{array}{c} 0\\ 0\end{array}\right) , \left( \begin{array}{cc} 1 & \rho _H \\ \rho _H & 1\end{array}\right) \right\} . \end{aligned}$$

For $j\in (f,m)$ we generated the binary parameter $\theta _j=I(\theta ^*_j>0)$, so that $\theta _j=0$ and $\theta _j=1$ correspond to the null and alternative hypothesis, respectively, for test *j*. The parameter $\rho _H$ is thus the tetrachoric correlation between $\theta _f$ and $\theta _m$. For $j\in (f,m)$ we generated population means as $\beta _j=0$ if $\theta _j=0$, and $\beta _j\sim N(0,1)$ if $\theta _j=1$. Finally, we generated $n=100$ paired observations $(Y_{fi},Y_{mi})$, $i=1 \ldots n$, from

$$\begin{aligned} \left( \begin{array}{c} Y_{fi}\\ Y_{mi}\end{array}\right)\sim &  N\left\{ \left( \begin{array}{c} \beta _f\\ \beta _m\end{array}\right) ,\left( \begin{array}{cc} 1 & \rho _Y \\ \rho _Y & 1\end{array}\right) \right\} . \end{aligned}$$

The parameter $\rho _Y$ is the conditional correlation between $Y_{fi}$ and $Y_{mi}$, given $(\beta _f,\beta _m)$, and thus determines the degree of dependence between the two observations in the pair.

For fixed values of $\rho _H$ and $\rho _Y$ we generated 300 samples from the model above. For each sample we stored the value of $\theta _j$ and computed the sample mean $\bar{Y}_j=\sum _{i=1}^nY_{ij}/n$, for $j\in (f,m)$. We used $\bar{Y}_j$ to test the null hypothesis $\theta _j=0$, by computing the usual *p* value $X_j=2\{1-\Phi (\bar{Y}_j\sqrt{n})\}$, for $j\in (f,m)$, where $\Phi$ is the standard normal distribution function. Using the 300 values of $X_f$ and $X_m$ we estimated the correlation between $X_f$ and $X_m$. We also regressed $\theta _f$ on $X_f$ and $X_m$ with the logistic model

$$\begin{aligned} \text {logit}\{p(\theta _f=1|X_f,X_m)\}=\beta _0+\beta _fX_f+\beta _mX_m. \end{aligned}$$

In this model, the parameter $\beta _m$ measures the conditional association between $\theta _f$ and $X_m$, given $X_f$. We remind the reader that $\theta _f=1$ is the alternative hypothesis. Thus, negative values of $\beta _f$ and $\beta _m$ means that large *p* values are associated with the null hypothesis and small *p* values are associated with the alternative hypothesis, as we would expect. We repeated the whole procedure to obtain 1000 estimates of $cor(X_f,X_m)$ and $(\beta _0,\beta _1,\beta _2)$, which we subsequently averaged.

The simulation described above was repated over a grid of values for $(\rho _H,\rho _Y)$. The contour plot in Fig. 4 shows the average estimate of $cor(X_f,X_m)$ as a function of $(\rho _H,\rho _Y)$. As expected, $cor(X_f,X_m)$ increases with both $\rho _H$ and $\rho _Y$. Figure 5 shows the average estimate of $\beta _m$ as a function of $(\rho _H,\rho _Y)$. When $\rho _H=\rho _Y=0$, $\beta _m$ is equal to 0 as well. We observe that $\beta _m$ decreases as $\rho _H$ increases. This is expected; the stronger correlation between $\theta _f$ and $\theta _m$, the stronger evidence does a small *p* value $X_m$ provide for $\theta _m=1$, and thus indirectly also for $\theta _f=1$. However, in Fig. 5 we observe that $\beta _m$ increases as $\rho _Y$ increases, and becomes positive when $\rho _Y$ is large and $\rho _H$ is small. This counter-intuitive feature is due to conditioning on the collider $X_f$, as discussed in Section ‘The Bayesian framework for dependent data’.

## Appendix 3: An example of a formal implementation of the Bayesian framework

Consider a pre-post-test study where an outcome is measured on a group of subjects during an unexposed period, and then again on the same subjects during an exposed period. The aim is to assess the exposure-outcome association within *J* strata defined by levels of a categorical variable. For simplicity we assume that all strata contain an equal number of *n* subjects. Let *Y* be the difference in the pre-post outcomes for a given subject, and let *Z* be the categorical variable that we wish to stratify on. Thus, the research question concerns the population means $\beta _j=E(Y|Z=j)$ for $j=1, \ldots ,J$. Under the null hypothesis of no exposure effect in stratum *j*, $\beta _j=0$.

Below we illustrate a Bayesian analysis of such data, and contrast with a standard frequentist analysis. The R code below requires the packages forestplot and bayesm. We load these as usual, by typing

To ensure that the results are reproducible we set the seed:

We simulate data under the ‘global’ null hypothesis $\beta _1= \cdots =\beta _J=0$ assuming that $Y|Z \sim N(0,1)$, for $n=10$ and $J=20$, with the code

Let $Y_{ij}$ be the value of *Y* for subject *i* in stratum $Z=j$. A standard frequentist analysis would estimate $\beta _j$ as $\hat{\beta }_j=\sum _{i=1}^nY_{ij}/n$ together with the 95% confidence interval $\hat{\beta }_j\pm t_{0.975,n-1}se_j$, where $t_{0.975,n-1}$ is the 0.975-quantile in the *t*-distribution with $n-1$ degrees of freedom, and $se_j=\sqrt{\frac{\sum _{i=1}^n(Y_{ij}-\hat{\beta }_j)^2}{(n-1)n}}$. We compute these estimates and confidence intervals as  and create a forest plot for the results:

Figure 6 shows the forest plot. We observe that, out of the 20 estimates, one is significantly different from 0 (i.e. the 95% confidence interval excludes 0) as expected.

Define $\beta =(\beta _1, \ldots ,\beta _J)$. To carry out a Bayesian analysis we use the runireg function from the bayesm package. This function uses MCMC sampling to generate random draws from the posterior distribution of $\beta$, under the model

$$\begin{aligned} Y\sim &  N(\beta X, \sigma ^2)\\ \beta\sim &  N(\mu , \sigma ^2A^{-1})\\ \sigma ^2\sim &  (\nu \times ssq)/\chi ^2_{\nu } \end{aligned}$$

By default, $E(\beta )=\mu$ is set to 0. The model allows for a correlation between the elements of $\beta$, by specifying a non-diagonal matrix $A^{-1}$. We refer to the manual for runireg for details.

We define the design matrix *X*, which specifies what stratum each subject belongs to, as

We first consider the extreme case where the elements of $\beta$ are considered a priori independent; $\text {cor}(\beta _j,\beta _k)=0$. We thus specify $A^{-1}$ as

We fit the Bayesian model as

The function runireg returns a list, which, among other things, contains 10,000 draws from the posterior distribution of $\beta$ in a matrix named betadraw. We compute the posterior estimates (medians) and 95% credible intervals as

and create a forest plot for the results:

Figure 7 shows the forest plot. We observe that the results are very similar to the results from the frequentist analysis, for all strata. In particular, the significant estimate in the frequentist analysis remains ‘significant’ (i.e. the 95% credible interval excludes 0) in the Bayesian analysis.

We next consider the case where the elements of $\beta$ are considered a priori strongly dependent; $\text {cor}(\beta _j,\beta _k)=0.95$. We thus run the code

Figure 8 shows the forest plot. We observe a striking difference between Figs. 7 and 8; the estimates are much closer to each other in Fig. 8, which is a result of the ‘shrinkage’ due to the assumed correlation between parameters. The significant estimate in the frequentist analysis is no longer ‘significant’ in the Bayesian analysis, since it has been ‘pulled towards 0’ by the other 19 estimates.

To further illustrate how the a priori correlation between parameters ‘shrinks’ the estimates toward each other, we re-ran the Bayesian analyses above for a range of a priori correlations between 0 and 0.95. For each value of the correlation we computed the sample variance of the estimates $(\hat{\beta }_1, \ldots , \hat{\beta }_J)$ as $\sum _{j=1}^J(\hat{\beta }_j-m)^2/(J-1)$, where $m=\sum _{j=1}^J\hat{\beta }_j/J$. Figure 9 shows the results. We observe that the sample variance of $(\hat{\beta }_1, \ldots , \hat{\beta }_J)$ decreases dramatically as $\text {cor}(\beta _j,\beta _k)$ increases.
